# Effects of Combined Anti-Hypertensive and Statin Treatment on Memory, Fear Extinction, Adult Neurogenesis, and Angiogenesis in Adult and Middle-Aged Mice

**DOI:** 10.3390/cells10071778

**Published:** 2021-07-14

**Authors:** Seungwoo Yoo, Matthew Stremlau, Alejandro Pinto, Hyewon Woo, Olivia Curtis, Henriette van Praag

**Affiliations:** 1 Stiles-Nicholson Brain Institute and Charles E. Schmidt College of Medicine, Florida Atlantic University, Jupiter, FL 33458, USA; yoos@health.fau.edu (S.Y.); apinto2014@fau.edu (A.P.); hyewonwoo@gmail.com (H.W.); ocurtis2017@fau.edu (O.C.); 2 National Institute on Aging (NIA), Baltimore, MD 21224, USA; stremlau@gmail.com

**Keywords:** atorvastatin, captopril, memory, anxiety, fear conditioning, adult neurogenesis, angiogenesis

## Abstract

Hyperlipidemia and hypertension are modifiable risk factors for cognitive decline. About 25% of adults over age 65 use both antihypertensives (AHTs) and statins to treat these conditions. Recent research in humans suggests that their combined use may delay or prevent dementia onset. However, it is not clear whether and how combination treatment may benefit brain function. To begin to address this question, we examined effects of atorvastatin, a 3-hydroxy-3-methylglutaryl coenzyme A (HMG-CoA) reductase inhibitor, and Captopril, an angiotensin-converting enzyme inhibitor (ACEI), administration on memory function, anxiety-like behavior, adult hippocampal neurogenesis and angiogenesis in adult and middle-aged male C57Bl/6J mice. In adult mice (3-months-old) combination (combo) treatment, as well as administration of each compound individually, for six weeks, accelerated memory extinction in contextual fear conditioning. However, pattern separation in the touchscreen-based location discrimination test, a behavior linked to adult hippocampal neurogenesis, was unchanged. In addition, dentate gyrus (DG) neurogenesis and vascularization were unaffected. In middle-aged mice (10-months-old) combo treatment had no effect on spatial memory in the Morris water maze, but did reduce anxiety in the open field test. A potential underlying mechanism may be the modest increase in new hippocampal neurons (~20%) in the combo as compared to the control group. DG vascularization was not altered. Overall, our findings suggest that statin and anti-hypertensive treatment may serve as a potential pharmacotherapeutic approach for anxiety, in particular for post-traumatic stress disorder (PTSD) patients who have impairments in extinction of aversive memories.

## 1. Introduction

With aging the risk of memory loss increases [1]. Hyperlipidemia and hypertension are precursors for cognitive decline that can be ameliorated with lifestyle changes such as diet, exercise, and medications [2,3,4,5,6]. While there is evidence that statins, HMG-CoA reductase inhibitors [7], and AHTs [8,9,10] are separately associated with cognitive benefits, recent research suggests that combined treatment with an angiotensin converting enzyme inhibitor (ACEI) and a statin substantially reduces the odds of dementia [11,12]. However, there are also studies that show no benefit of combination therapy for cognitive decline in the elderly [13]. Indeed, multiple factors may influence the effects of antihypertensive and lipid lowering therapy on human brain function such as age, baseline blood pressure, cholesterol, and blood glucose levels [14]. Animal models may provide insight as to whether these compounds affect memory and mood, and the possible underlying mechanisms.

Evidence for effects of statins on memory and mood-related behaviors, derives mainly from animal models of neurological injury or disease, both in vivo [15,16,17,18,19] and in vitro [20]. For instance, in rats subjected to traumatic brain injury (TBI) [15] or hypertensive rats [21] performance in the Morris water maze was improved by atorvastatin or rosuvastatin, respectively. Treatment of Fmr1 knockout rats with lovastatin prevented development of memory deficits [22]. Atorvastatin protected against anxiety- and depressive-like behavior during aging [23], in a rat model of epilepsy [24] and following lipopolysaccharide (LPS) treatment [17]. Research into potential underlying mechanisms suggest statins regulate brain-derived neurotrophic factor (BDNF) levels [17], cerebral vascularization [15], and adult hippocampal neurogenesis [16,18,19,25].

Similar to statins, studies in rodents pertaining to effects of ACEIs on memory function, have generally been conducted in models of neurological disease or injury. For instance, ACEIs captopril and ramipril attenuated scopolamine-induced deficits on memory tests in mice [26,27]. Captopril treatment also improved LPS-induced spatial memory impairment in rats [28,29], and reduced neuropathology in the Tg2576 Alzheimer’s disease mouse model [30] and in aged rats subjected to chronic mild stress [31]. Additionally, captopril ameliorated chronic stress-induced depressive-like behaviors and reversed synaptic loss in mice [32]. Furthermore, in addition to lowering blood pressure and improving cerebral blood flow, ACEIs reduce inflammation and oxidative stress [33,34,35]. Moreover, ACEI attenuated the decrease in adult hippocampal neurogenesis resulting from x-radiation [36], and this effect was enhanced in combination with atorvastatin [37].

Thus, the majority of statin and antihypertensive studies pertaining to brain function have been conducted in rodent models of injury and disease. It remains unclear, however, whether and how combined statin and anti-hypertensive treatment may affect the brain during normal aging and in adulthood. Therefore, this study examined how treatment with atorvastatin and captopril affects learning and memory, anxiety-like behaviors, adult hippocampal neurogenesis and angiogenesis in adult and middle-aged male mice.

## 2. Materials and Methods

### 2.1. Subjects

Middle-aged male C57BL/6J mice (NIA aging mouse colony), 10-months-old at the beginning of the experiment (*n* = 20), were housed individually in standard cages with a standard day/night cycle: lights were switched on at 6:00 AM and off at 6:00 PM. Mice were divided into control (*n* = 10) and combo groups (atorvastatin and captopril, *n* = 10). Mice were given ad libitum access to water and food. Mice underwent behavioral testing at 12 months of age in the open field arena and Morris water maze. The experimental protocols were approved by the National Institute on Aging Institutional Animal Care and Use Committee, and all animals were maintained according to the National Institute of Health guidelines.

Adult male C57BL/6J mice (Jackson Laboratory, Bar Harbor, ME, USA), 14 weeks old at the beginning of experiment (*n* = 58) were housed individually in standard cages with a standard day/night cycle: lights were switched on at 7:30 AM and off at 9:30 PM. Mice were divided into control (*n* = 9), atorvastatin (*n* = 9), captopril (*n* = 10), and combo (atorvastatin and captopril, *n* = 10) groups for the open field and fear conditioning tests. Mice were given ad libitum access to water and food. Another set of adult mice was divided into control (*n* = 10) and combo groups (atorvastatin and captopril, *n* = 10) for the pattern separation task. These mice were given ad libitum access to water but were food-restricted to 85% of free feeding weight for the behavioral experiments. All animal-use procedures were conducted in accordance with the guidelines required by the National Institutes of Health Guide for the Care and Use of Laboratory Animals. Florida Atlantic University’s Institutional Animal Care and Use Committee approved all procedures prior to the onset of the experiments.

### 2.2. Drug Administration

*Atorvastatin*. Atorvastatin (Sigma, PHR1422) was dissolved first in dimethyl sulfoxide (DMSO, 50 mg/mL; Sigma, 34869) and then added to water (0.2 mg/mL) for the adult and middle-aged mouse groups. Sucrose was added at a final concentration of 2% in all solutions to mask any adverse tastes. For the control group DMSO was not added to the drinking water, which may represent a potential confound. The average daily dose of atorvastatin was 0.88 mg based on an average drinking volume of 4.4 (±0.3) mL per mouse per day in middle-aged mice, and 1.10 mg based on an average liquid intake of 5.7 (±0.7) mL per day in adult mice.

*Captopril*. Captopril (Sigma, C4042 for adult mice; Sigma, PHR1307 for middle-aged mice) was dissolved in 0.9% saline and administered by intraperitoneal (i.p.) injection in a dosage of 10 mg/kg. Atorvastatin and captopril were administered daily for a week at a time for two cycles of treatment, for a total of six weeks of treatment.

*BrdU.* To label dividing cells that can develop into new neurons in the adult dentate gyrus of the hippocampus the thymidine analog bromodeoxyuridine (BrdU) was injected intraperitoneally [38]. Specifically, at the onset of compound treatment, mice were given a daily i.p. injection of BrdU (Sigma, B5002), 50 mg/kg, for the first 10 days. BrdU was dissolved in 0.9% saline, 10 mg/mL concentration; filtered sterile at 0.2 μm.

*Sequential treatment*. We chose this treatment schedule because it allows for optimal delivery of each compound, and may minimize potential unwanted side effects that could result from additive or synergistic effects of simultaneous administration of the compounds. The treatment schedule we used was also aimed at reducing injection stress in the mice. While statins can be administered in drinking water [23,24] or in the diet [22], captopril is given by injection in mice [39,40,41] and in rats [28,29,42].

### 2.3. Behavioral Experiments

*Open field test.* Spontaneous locomotor activity and anxiety level were evaluated by using an infrared photobeam-controlled open-field testing chamber (Version 4.0, Med Associates, St. Albans, VT, USA; height 20.3 cm, width 27.3 cm, depth 27.3 cm). The center zone was defined as an area of 14.29 cm × 14.29 cm. Mice were placed in the square open field and explored freely for 60 min. Animal’s movement was detected by three 16-beam IR arrays installed on X, Y, and Z axes and the time and distance in the center and periphery of the open field were automatically recorded by Activity Monitor software.

*Morris water maze test.* Mice were trained in the water maze with 4 trials a day for 9 days [43]. The platform (15 cm × 15 cm) was hidden 1 cm below the surface of a pool (160 cm diameter) filled with opaque water (26 °C) mixed with white nontoxic paint. Visual cues were attached on the walls of the experimental room. The hidden platform was located in the northwest (NW) quadrant of the pool. Mice were gently placed on the surface of water and the starting point was randomly changed across trials by the experimenter. Each trial ended when a mouse had found the platform or when 60 s had passed, whichever came first. At the end of each trial, mice were allowed to rest on the platform for 10 s (inter-trial interval). The latency and distance to reach the platform were recorded semi-automatically by a video tracking system (AnyMaze, Stoelting). Probe trials were conducted 4 h and 24 h after completion of training to test spatial memory retention. The platform was removed for the 60 s probe trials. Time spent swimming in each quadrant, the number of platform crossings and latency to the first platform crossing were recorded semi-automatically by a video-tracking system (AnyMaze, Stoelting).

*Fear conditioning test.* Contextual fear memory was tested by using the near-infrared video fear conditioning system (MED Associates Inc., Fairfax, VT, USA) which is composed of four fear-conditioning chambers. Each chamber consists of transparent Plexiglas walls and top with brushed aluminum side frames and a floor of parallel stainless-steel rods which is connected to a shock generator. The tray located in the bottom of the chamber contains 1% liquinox solution (1% acetic acid for the tone-cued memory retrieval). The chamber has a speaker which delivers a tone and an overhead white light that illuminates the inside of a chamber throughout testing. Each chamber was placed inside a larger noise-attenuating cubicle (height 31.75 cm × width 71.12 cm × depth 59.69 cm) which includes a ventilation fan delivering background noise. Freezing behavior, a rodent’s natural response to fear [44], was measured by a near-infrared camera-tracking system (MED Associates, Georgia, VT, USA) mounted on the inside of the chamber. The tone-cued and contextual memory test were composed of four sessions across four days, and the extinction of contextual memory were tested 10 and 17 days, respectively, after the onset of the experiment. For each session, four mice were transferred to the procedure room and kept in their home cage for 10 min. Following 10 min, the individual mice were placed in the conditioning chambers, and trials began. Mice were removed from the chambers at the end of the trial. The inner surfaces of a chamber were thoroughly cleaned with 70% isopropyl alcohol after completion of each session. **Day 1 (Habituation)**: On the first day, mice were placed in the chamber and freely explored the inside of the chamber for 5.5 min. **Day 2 (Conditioning)**: Twenty-four hours later, mice were placed in the chamber. After 60 s, mice were given a conditioned stimulus (CS), a tone (5000 Hz, 90 dB tone), for 30 s, that was co-terminated with unconditioned stimulus (US), a foot shock (1 s, 0.5 mA). Thereafter, the paired CS-US was repeated two more times with a 90 s inter-stimulus interval. **Day 3 (Auditory-cued memory and Contextual memory)**: Twenty-four hour later, tone-cued memory retrieval was tested. In the tone-cued test, the original chamber was modified by inserting an arch-shaped plastic panel inside of the chamber, and a blue semitransparent plastic film was put on the top of the chamber to provide an altered-visual environment. In addition, a 1% acetic acid solution was put in the bottom of the tray to provide differential olfactory information to mice. At the onset of testing, the animal was placed in the modified chamber for 60 s and the CS was presented for 30 s followed by 60 s inter-stimulus intervals without shock. The CS was repeated two more times. One hour later, extinction of contextual fear memory was subsequently tested in the original condition that was used in the habituation and conditioning sessions. Mice were allowed to move freely in the chamber during 5.5 min and no tone or shock were presented. **Day 10 and 17 (Extinction of contextual memory)**: On day 10 and 17 after completion of the contextual memory test on day 3, extinction of contextual memory was tested in the original condition during 5.5 min without tone and shock presentation. In the fear conditioning test, freezing is defined as the lack of activity except for respiration, lasting for more than 0.6 s. The software automatically measures freezing behavior, with the video capture rate set at 30 frames per second (fps) resulting in freezing being recorded after less than 20 pixels of motion per frame over the time course of 18 frames [45].

*Touchscreen-based pattern separation test.* The testing apparatus for the location discrimination (LD) task is composed of a sound attenuating cubicle (width 56.5 cm × depth 53.2 cm × height 54.5 cm) and a touch screen chamber (width at feeder 4.6 cm × width at screen 23.8 cm × depth 17 cm × height 23 cm) which consisted of a black Plexiglas trapezoidal wall, touch screen installed with infrared sensor (WhiskerServer Controller), a perforated-metal floor, reward area, a liquid pump feeder, a speaker with tone generator, a tray light and a house light. A black Plexiglas “mask” panel, which includes two rows of six square windows (2.5 cm × 2.5 cm, equally spaced 1 cm apart) located 1.6 cm from the bottom of mask panel was inserted in front of the touch screen that presented white stimuli on the 6 windows at the bottom of the mask panel. When an animal touches the white stimulus coming from one of the 6 windows, the software (ABET II Touch software) counted and saved its activity automatically. **Pre-training (6 weeks)**: Mice (*n* = 20) were given ad libitum access to water and food-restricted to 85% free feeding weight from 3 days before the beginning of the LD task throughout the study. Habituation: For the first 2 days of shaping, mice were placed in the touch screen chambers for 20 min each day to become accustomed to the chamber environment. In the chamber the screen was turned off, the house light on, and the reward trough was filled with vanilla-flavored liquid reward (Ensure, Abbott). **Pavlovian training (Initial touch training)**: On day 3, animals underwent Pavlovian initial touch training. The stimulus (a white square) was randomly displayed in one of the bottom row windows and the rest of the windows were blank. The stimulus was not displayed in the same position for more than three times in a row. When the mouse touched the stimulus, a drop of liquid reward and a tone were delivered with illumination of the food tray light. Entry to collect the reward turned off the tray light and triggered the inter-trial interval (ITI, 10 s) for the next trial. The duration of one trial was from the onset of the stimulus to the end of the ITI. Following the ITI, the next stimulus was displayed in one of the bottom row windows. Mice were trained until they reached a criterion of 30 trials in 1 h. **“Must touch” stage**: A trial began with the stimulus presented randomly in one of the windows of the bottom row, while the other windows were left blank. When the mouse touched the stimulus, the tone, the tray light and a drop of reward were delivered followed by a 10 s ITI. After the ITI, the next trial followed. A touch to a blank window was regarded as “incorrect” and no tone or reward were provided. Mice were trained until they reached a criterion of 30 trials in 1 h. **“Must initiate” stage**: The stimulus was presented in the same way as in the “Must touch” stage and a touch to the stimulus elicited the tone and reward response with the tray light. Following a 10 s ITI after obtaining the reward, mice had to poke their nose into the reward trough again to initiate the next trial. Once a mouse had reached a criterion of 30 trials in 1 h, the next stage began. **“Punish Incorrect” stage**: The stimulus and reward were given in the same way as described above. However, if a mouse touched the blank window, instead of the white stimulus window, the house light in the ceiling of the chamber was inverted for a time-out period (5 s) and no reward was provided. After the time-out period finished, the house light was reverted and the 30-s ITI period began. Mice were trained until a criterion of 30 trials in 30 min with 23 correct trials (85% correct out of total trials performed) for which two consecutive days was reached. **Compound administration (6 weeks)**: When the pre-training sessions were completed, mice were treated with compounds for six weeks as scheduled in each group. For the first 10 days of compound treatment, mice were given a daily i.p. injection of BrdU (50 mg/kg). **Task training (21 days)**: At the beginning of each trial, two white square stimuli were presented at positions 2 and 5 out of the six windows, with position 1 being the left-most window. For each trial, only one of two stimuli was reinforced with both tone and liquid reward, and the correct side of the stimuli was randomly counterbalanced between mice. When an animal reached the performance criterion, seven trials out of eight trials correct, the reward-reinforced stimulus was reversed, in which the former incorrect window became the reinforced stimulus and vice versa. This training lasted for 60 trials or until eight reversals were reached, whichever came first. The next day, animals were trained in the same way and the initially correct window was the same one as in the last session of the day before. Mice were trained until they reached a performance criterion that achieved the acquisition, 7 out of 8 trials correct, for 3 out of 4 days in a row. **Probe trial (12 days)**: For the probe trial sessions, either a small or a large separation condition was presented and counterbalanced between mice. In the large separation trial, two stimuli were simultaneously displayed in windows 1 and 6, the most left and right side of 6 windows respectively, and the two stimuli were displayed in window 3 and 4 in the small separation trial. Over 60 min, unlimited trials were provided. Each separation was alternated every 2 days over 12 days of testing. For instance, if a mouse began with the small separation, this condition continued for 2 days, first and second days, and then the big separation was presented on the third and fourth days. This pattern was repeated for 12 days of testing is completed. The number of trials to criterion (7 out of 8 trials correct), the number of trials completed, and the number of reversals were used for the data analysis. If a mouse had not reached the acquisition criterion in either the small or large separation in the probe trial, the total number of trials performed within that session + 8 was used as this would have been the minimum amount of trials to reach the criterion [46].

### 2.4. Histology/Immunohistochemistry

*Tissue preparation.* After completion of behavioral testing, animals were given an overdose of isoflurane anesthetic (Abbott) for aged mice or an overdose of drug injection (ratio, 1:1.4:6.4, Xylazine, Ketamine and Saline, respectively) for adult mice, and perfused transcardially with 0.9% saline at room temperature (RT) followed by ice cold 4% paraformaldehyde in 0.1 M PBS. After perfusion, brains were dissected, post-fixed for 24 h, and subsequently equilibrated in 30% sucrose. Sequential coronal sections (40 μm) were taken using a freezing microtome (HM450, ThermoFisher Scientific, Wyman St. Waltham, MA, USA) through the rostral-caudal extent of the brain and stored in phosphate buffered glycerol at −20 °C.

*BrdU/NeuN staining.* A one-in-six series of free-floating sections (40 μm) was washed in tris-buffered saline (TBS), incubated in 2 N HCl at 37 °C for 30 min to denature DNA, and then neutralized in 0.1M borate buffer. Following washes in TBS sections were co-incubated for 72 h in primary antibodies, anti-rat BrdU (1:500, Abcam) and the neuronal marker anti-mouse NeuN (1:500, Abcam, Cambridge, UK) in TBS with 3% donkey serum and 0.1% Triton X-100 (TBS^++^), at 4 °C. Thereafter, sections were co-incubated with donkey anti-rat Alexa Fluor 488 (1:250, Abcam) and donkey anti-mouse Cy3 (1:250, Jackson ImmunoResearch, West Grove, PA, USA) in TBS^++^ for 2 h, at room temperature. 

*CD31 staining.* A one-in-six series of free-floating sections (40 μm) was washed in TBS and pre-incubated with 30% H2O2 for 30 min to quench endogenous peroxidases. Next, sections were washed in TBS again and incubated in rat anti-CD31 (1:500, BD Pharmingen) in TBS^++^ overnight, followed by staining with a biotinylated donkey anti-rat secondary antibody (1:250, Jackson ImmunoResearch Laboratories) in TBS^++^ for 4 h, at room temperature. Staining was completed using the ABC peroxidase complex (ABC Kit, Vector Laboratories, Burlingame, CA, USA) and the chromogen 3,3′-diaminobenzidine (DAB, Sigma-Aldrich, St. Louis, MO, USA).

### 2.5. Quantification and Statistical Analysis

*Adult hippocampal neurogenesis*. Sections, one-in-six series (240 μm apart) were imaged at 20× throughout the rostral-caudal extent of DG with a confocal microscope (Nikon, A1R), with projections of 12-15 *Z* planes taken at 2-μm intervals. Total BrdU^+^ cell numbers were obtained by multiplying by the counts in the series by six. Thirty BrdU^+^ cells in the dentate gyrus per animal were randomly selected and imaged for neuronal phenotype analysis. The percentage of BrdU^+^/NeuN^+^ cells was calculated [47].

*Angiogenesis in DG.* The total volume of blood vessels covering the region of interest, the DG, was quantified using IMARIS 9.6.0 software. First, the raw images of the brain sections were converted to grayscale, and then the contrast of the grayscale images was inverted. Next, to define the volume and intensity of blood vessels, the threshold filtering algorithm provided by IMARIS software was applied to all images. Finally, the percentage of DG blood vessels was calculated for each image. The region of interest was defined as the DG granule cell layer, hilus, and molecular layer, distinguished by the hippocampal fissure. The experimenter who was blind to the classification of the treatment groups traced the region of interest.

*Statistical analysis.* All statistical analyses were carried out using Statview (Abacus Corporation). The analyses performed for the experiments are as follows:

- The open field test: unpaired *t*-tests between the groups in middle-aged mice, and one-way ANOVA for adult mice (Group × Time) and (Group × Distance).
- The Morris water maze test: one-way ANOVA with repeated measures for acquisition of latency, path length, and swim speed (Group × Days) and probe test (Group × Time (4 h, 24 h)). Within-group analyses: one-way ANOVA with repeated measures (Quadrant × Time (4 h, 24 h)).
- Adult hippocampal neurogenesis and angiogenesis: Unpaired *t*-tests (middle-aged mice). Comparison between adult and middle-aged mice: two-way ANOVA (Age × Treatment).
- Pattern separation task: Two-way ANOVA (Group × Task level).
- Fear conditioning test: One-way ANOVA with repeated measures: Habituation (Group × Time), conditioning (Group × Events), tone-cued memory (Group × Events), and memory extinction (Group × Days). Bonferroni/Dunn was used for post-hoc comparisons. The number of subjects per group was based on our previous publications [47,48]. All data are presented as means ± standard error of the mean (SEM).

## 3. Results

### 3.1. Open Field and Morris Water Maze Performance in Middle-Aged Mice

**Figure 1:**
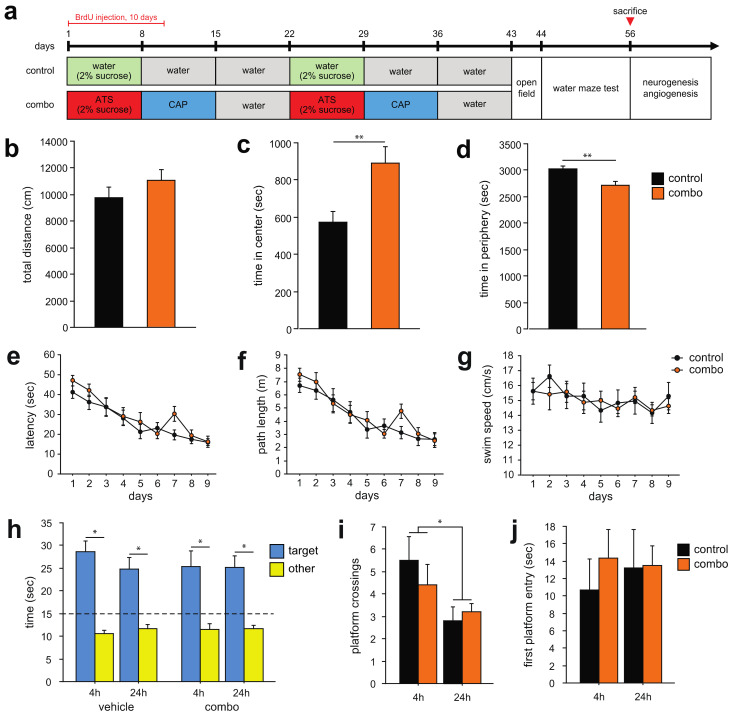
Effects of compound treatment on locomotion, anxiety and spatial memory in middle-aged mice. (**a**) Compound treatment and behavioral testing schedule in middle-aged mice. At the onset of the experiment, each compound was administered to mice daily for a week at a time for two cycles, a total of six weeks of treatment. (**b**) There was no difference in locomotor distance between the groups. (**c**) The combo group spent significantly more time in the center of the arena than the control group and (**d**) less time in the periphery, indicating that compound treatment reduces anxiety. (**e**–**j**) Mice were trained in the water maze with 4 trials a day for 9 days. There was no difference between the groups in (**e**) latency and (**f**) distance to the platform or (**g**) swim speed. (**h**) In the probe trials 4 and 24 h after the last acquisition session, both groups spent significantly more time in the target area than the other quadrants. (**i**) The number of platform crossings did not differ between the groups, however, both groups displayed less crossings in the 24 h as compared to 4 h probe trial. (**j**) Latency to the first platform crossing did not differ between the groups. Atorvastatin, ATS; captopril, CAP; bromodeoxyuridine, BrdU. * *p* < 0.05, ** *p* < 0.01. The dashed line in (**h**) represents chance level in the probe test.

Following combined atorvastatin and captopril (combo) treatment, open field behavior was evaluated in the middle-aged mice (Figure 1a). Ambulatory time and distance in the center and periphery of the open field arena were measured. There was no difference in the total locomotor distance (center and periphery) between the groups (t_(18)_ = 1.2, *p* = 0.23, Figure 1b). However, the combo group spent significantly more time in the center and less time in the periphery of the arena than the control group (t_(18)_ = 3.0, *p* = 0.007; Figure 1c,d). Thus, combo treatment may reduce anxiety in middle-aged mice.

Next, mice were trained in the Morris water maze [43]. There was no significant difference between the groups in the latency (F_(1,18)_ = 0.8, *p* = 0.55, Figure 1e) and path length (F_(1,18)_ = 0.7, *p* = 0.64, Figure 1f) to the platform, or in the average swim speed (F_(1,18)_ = 0.6, *p* = 0.69, Figure 1g). Four hours and 24 h after completion of the last training session, probe trials were performed. There was no significant interaction between the groups in the time spent in the target quadrant over the probe trials (F_(1,18)_ = 1.5, *p* < 0.23). Within-group analysis showed a significant preference for the target quadrant as compared to the other quadrants in both groups in the probe trials (F_(3,9)_ = 22.3, *p* < 0.0001, control; F_(3,9)_ = 11.1, *p* < 0.0001, combo; Figure 1h). There was no difference between the groups in the number of platform crossings (F_(1,18)_ = 1.1, *p* = 0.3, Figure 1i). However, platform crossings were significantly less in the 24 h as compared to 4 h probe trial (F_(1,18)_ = 7.6, *p* < 0.012; Figure 1i). There was no difference between the groups in the latency to the first platform crossing (F_(1,17)_ = 0.2, *p* = 0.64, Figure 1j). These results indicate that combo treatment does not affect spatial learning and memory in middle-aged mice.

### 3.2. Adult Hippocampal Neurogenesis and Angiogenesis in Middle-Aged Mice

**Figure 2:**
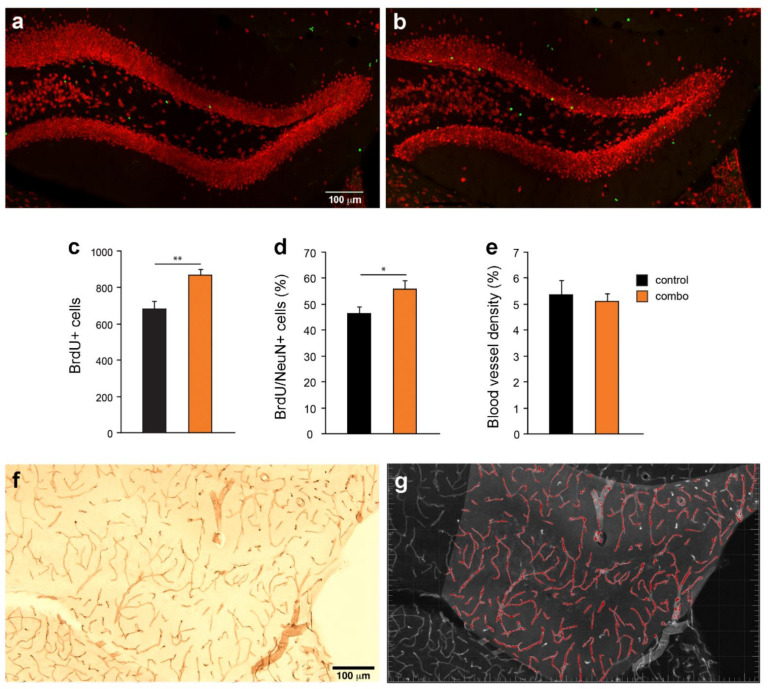
Effects of compound treatment on adult hippocampal neurogenesis and angiogenesis in the dentate gyrus in middle-aged mice. (**a**,**b**) Photomicrographs show double-labeling for BrdU (green) and NeuN (red) of coronal hippocampal sections derived from (**a**) control and (**b**) combo-treated mice. (**c**) The number of BrdU^+^ cells significantly increased in the combo as compared to the control group. (**d**) The percentage of BrdU^+^/NeuN^+^ cells was significantly higher in the combo than the control group. (**e**) The density of DG blood vessels did not differ between the groups. (**f**) Photomicrograph of blood vessel labeling with CD31. (**g**) Processed image (blood vessels are identified in red) for vasculature analysis. * *p* < 0.05, ** *p* < 0.01.

Dentate gyrus BrdU^+^ and BrdU^+^/NeuN^+^ cells were quantified (Figure 2a–d). In middle-aged mice, treatment significantly increased the number of BrdU^+^ cells (t_(15)_ = 3.8, *p* = 0.0016, Figure 2c) and the percentage of BrdU^+^/NeuN^+^ cells (t_(15)_ = 2.3, *p* = 0.03, Figure 2d). DG blood vessel density (Figure 2e–g), did not differ between the groups (t_(12)_ = 0.4, *p* = 0.64, Figure 2e). Thus, combined treatment increases adult hippocampal neurogenesis but not angiogenesis (Table 1).

### 3.3. Pattern Separation, Adult Hippocampal Neurogenesis, and Angiogenesis in Adult Mice

**Figure 3:**
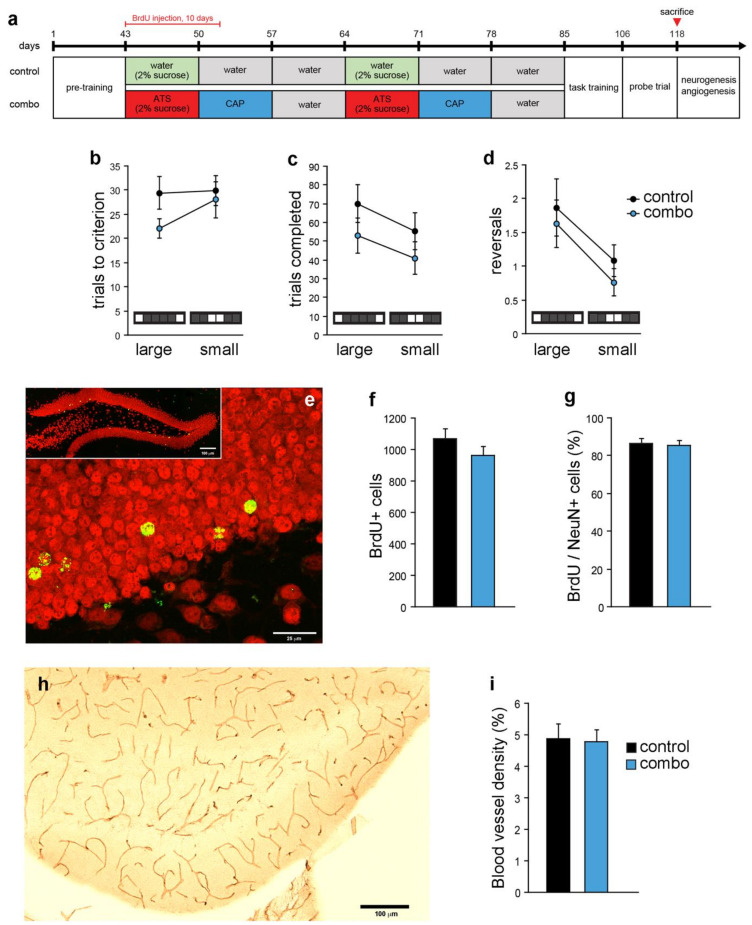
Effects of compound treatment on pattern separation, adult hippocampal neurogenesis and angiogenesis in the dentate gyrus in adult mice. (**a**) Compound treatment and touchscreen-based pattern separation test schedule in adult mice. After mice reached the performance criterion in pre-training, each compound was administered to mice daily for a week at a time, for two cycles. Treatment lasted a total of six weeks and both groups were given a daily i.p. injection of BrdU for the first 10 days. Following compound treatment, mice were trained again in task training until they reach performance criterion. The days to complete the task training did not differ between groups. Thereafter, both groups were tested in the probe trials for 12 days. (**b**) In the number of trials to criterion, 7 out of 8 trials correct within a session, there was no interaction between task level (small and large separation) and the groups. (**c**) Trials completed and (**d**) the number of reversals also did not differ between groups. (**e**) Photomicrograph of double-labeling for BrdU (green) and NeuN (red) in the DG. (**f**) There is no difference in the number of BrdU^+^ cells or (**g**) in the percentage of BrdU^+^/NeuN^+^ cells between the groups. (**h**) Photomicrograph of DG blood vessels stained with CD31. (**i**) DG blood vessel density did not differ between the groups. Atorvastatin, ATS; captopril, CAP; bromodeoxyuridine, BrdU; dentate gyrus, DG; NeuN, neuronal nuclei.

To evaluate whether compound treatment can affect the ability to discriminate between similar stimuli, adult mice (3-months-old) were pre-trained in the pattern separation task until they reached performance criterion prior to compound treatment (Figure 3a).

After six weeks of compound treatment, mice underwent task training (7 months old at the onset) until they reached a performance criterion for 3 out of 4 days in a row. The days to complete the task training did not differ between groups (data not shown, t_(17)_ = 0.8, *p* = 0.39). Following the task training, both groups were then tested in probe trials with either a large or a small separation between stimuli for 12 days. Analysis of the number of trials to criterion, 7 out of 8 trials correct within a session, showed that there is no interaction between the task level (small and large separation) and the treatment groups (F_(1,17)_ = 1.2, *p* = 0.28, Figure 3b). The number of trials completed (F_(1,17)_ = 0.08, *p* = 0.77, Figure 3c) and the number of reversals (F_(1,17)_ = 0.04, *p* = 0.84, Figure 3d) also did not differ between the groups. Thus, compound treatment in adult mice does not affect the pattern separation ability.

The effect of compound treatment on adult hippocampal neurogenesis was also evaluated (Figure 3e–g). BrdU^+^ cell number (t_(17)_ = 1.3, *p* = 0.18; Figure 3f) and the percentage of BrdU^+^/NeuN^+^ cells did not differ between the groups (t_(16)_ = 0.3, *p* = 0.7; Figure 3g). Comparisons between adult and middle-aged groups showed that new neuron number was lower in control middle-aged mice, consistent with an age-dependent decline in adult neurogenesis, and that combo treatment reversed this decrease (Table 1). In particular, comparisons across age groups showed that there was a significant interaction between age group and treatment (F_(1,32)_ = 9.596, *p* < 0.004), and a main effect of age (F_(1,32)_ = 25.956, *p* < 0.0001) for BrdU^+^ cells. In addition, there was a close to significant interaction (F_(1,31)_ = 4.034, *p* < 0.0534), and a significant main effect of age (F_(1,31)_ = 174.62, *p* < 0.0001) for the percentage of BrdU^+^/NeuN^+^ cells. There was no difference in DG blood vessel density between the adult combo and control groups (t_(18)_ = 0.1, *p* = 0.88; Figure 3h,i) or between adult and middle-aged mice (Table 1). Overall, these results show that combo treatment affects adult hippocampal neurogenesis, but not DG angiogenesis, in an age-dependent manner.

### 3.4. Open Field Behavior and Contextual Fear Memory in Adult Mice

**Figure 4:**
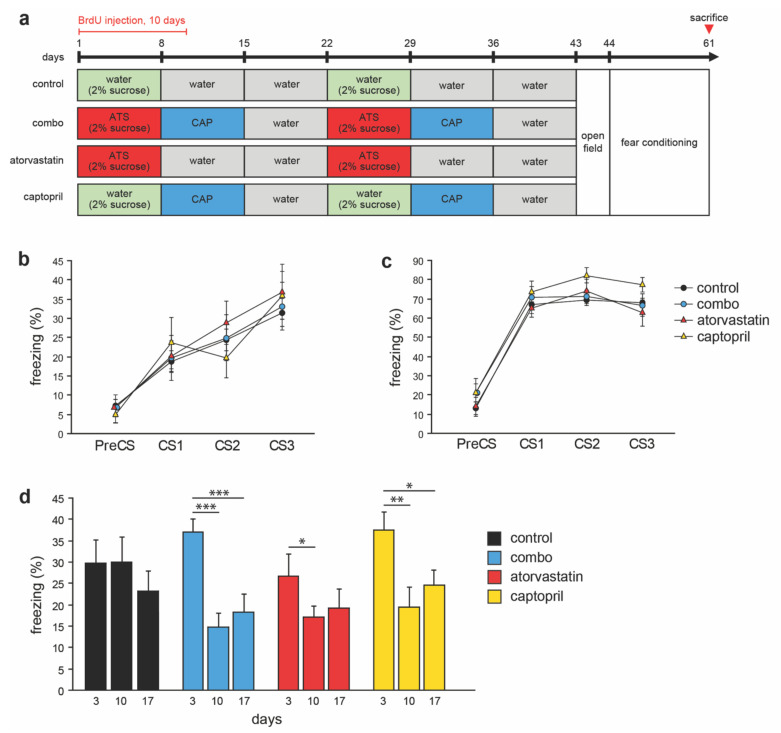
Effects of compound treatment on contextual fear memory in adult mice. (**a**) Compound treatment and behavioral testing schedule of the adult mice. At the start of the experiment, each compound was administered to mice daily for a week at a time, for two cycles. Treatment lasted for a total of six weeks. (**b**–**d**) Contextual fear conditioning testing: following habituation and conditioning sessions on the first and second day, respectively, tone-cued memory retrieval was tested on day 3. On the same day, contextual fear memory in the original environment was examined. Subsequently, memory extinction was tested on day 10 and 17. (**b**) No significant difference between the groups was found in conditioning session (PreCS, CS1, CS2, and CS3) or in (**c**) the tone-cued memory retrieval session. (**d**) There was a significant interaction between groups in freezing time during the extinction trials. Post-hoc comparisons showed that the combo, captopril and atorvastatin groups, but not the control group showed extinction of fear over time. Atorvastatin, ATS; captopril, CAP; bromodeoxyuridine, BrdU. * *p* < 0.05, ** *p* < 0.01, *** *p* < 0.001.

In a separate cohort of adult mice each group was given either atorvastatin, captopril, or combined treatment, followed by behavioral testing (the mice were 5-months of age at the onset of behavioral experiments) (Figure 4a). In the open field test, there were no differences in distance travelled (F_(3,34)_ = 0.7, *p* = 0.55) and in center and periphery time (F_(3,34)_ = 1.06, *p* = 0.37), between the groups (Table 2), indicating that neither individual nor combo treatment affects ambulatory movement and anxiety level in the open field test in adult mice.

In the fear conditioning test, on the first day, similar freezing levels in the habituation session were observed in the groups (F_(3,34)_ = 1.1, *p* = 0.34). On the second day, mice were trained in the conditioning session, in which the conditioned stimulus (tone) and unconditioned stimulus (foot shock) were provided. There was no interaction between the groups and events (PreCS, CS1, CS2 and CS3) in the conditioning session (F_(3,34)_ = 0.4, *p* = 0.89, Figure 4b). On day 3, mice were tested in the tone-cued memory session that provided the tone three times without shock in a new contextual environment. There was no interaction between the groups in the tone-cued memory retrieval session (F_(3,34)_ = 0.6, *p* = 0.73, Figure 4c). One hour later, 10 days and 17 days later mice were tested in the memory extinction sessions in the original environment used in the habituation and conditioning sessions. There was a significant interaction between time (day 3, day 10 and day 17) and groups (F_(3,34)_ = 2.6, *p* = 0.02) in the memory extinction sessions. Post-hoc analysis revealed a significant reduction over time in freezing in the combo (day 3 vs. day 10, *p* = 0.0002; day 3 vs. day 17, *p* = 0.0009), the captopril (day 3 vs. day 10, *p* = 0.001; day 3 vs. day 17, *p* = 0.01) and the atorvastatin (day 3 vs. day 10, *p* = 0.02) groups, but not in the control group (Figure 4d). These results suggest that combo, atorvastatin and captopril treatment increases memory extinction.

## 4. Discussion

This study examined whether combined treatment with atorvastatin and captopril could affect neurogenesis, angiogenesis, cognition, and anxiety-like behavior in adult and middle-aged wild-type mice. We found that in middle-aged mice combo treatment reduced anxiety-like behavior in the open field test but did not affect spatial memory. This treatment increased adult hippocampal neurogenesis, but not angiogenesis. In adult mice spatial pattern separation in the touchscreen, neurogenesis and angiogenesis were unchanged. Enhanced extinction in the contextual fear conditioning test, following combined treatment as well as with each compound separately was observed in the adult group. A role for statins in memory extinction has not been previously described. Overall, these compounds may benefit patients with anxiety disorders.

High systolic blood pressure and hypercholesterolemia in midlife are widely recognized risk factors for dementia [49]. A number of animal studies have provided evidence for beneficial effects of statins [15,17,23,50,51] and ACEI [28,29,52] on cognitive function and anxiety- and depression-like behaviors. However, in most previous studies, drugs were administered separately, and in genetically modified animals [50,51,52] or chronic disease models [15,17,23,28,29]. In our study we show that spatial memory was not affected in middle-aged mice. However, in the open field test these mice displayed reduced anxiety as evidenced by increased center time. It remains unclear whether individual administration of each compound would have similar effects on behavior in middle-aged mice, and whether different dosing paradigms might further enhance the observed effects or potentially result in detrimental outcomes [53].

The concomitant increase in adult neurogenesis in the middle-aged rats supports the positive effect of the combined treatment on anxiety-like behavior. Adult hippocampal neurogenesis is closely associated with cognition and mood [54]. Our findings are in agreement with the previous observations following statin treatment showing enhanced cell proliferation and neurogenesis in mouse models of brain injury [16,18,19,25]. With regard to ACEIs, there are equivocal reports. It has been reported that ramipril enhances neurogenesis following radiation injury but not under basal conditions [36,37]. Other researchers did not find an enhancement of adult neurogenesis in spontaneous hypertensive rats [55]. The combined treatment enhanced the survival of newly generated cells and resulted in an increase in the percentage of new cells that differentiated into neurons. While the enhancement of adult neurogenesis (~20%) is modest, it may contribute to the observed reduction in anxiety-like behavior [54]. Other effects of statin and ACEI treatments, such as reduced inflammation and enhanced BDNF levels, may also play a role [29,56] but were not examined in the present study. Vascularization was measured in the hippocampus as previous studies utilizing statins and anti-hypertensives indicated beneficial effects [15,35], but was not changed in the middle-aged mice. While our methods are consistent with previous studies [57,58,59], a potential limitation is that the blood vessels were not analyzed in 3D [60], which could have optimized the chances of detecting differences between groups.

Hippocampal neurogenesis is considered to play an important role in pattern separation [54,61], the ability to distinguish between closely related events or stimuli. A previous report indicated that simvastatin enhances adult hippocampal neurogenesis and Wnt signaling in 2-month-old mice C57Bl/6 mice [25]. Therefore, we investigated whether combined treatment affects spatial pattern separation and adult neurogenesis in adult mice. However, no differences were observed between control and treatment groups in either measure, nor did angiogenesis differ between the groups. A separate cohort of mice that was treated with the combination, or with each compound individually was tested in the contextual fear conditioning test, a memory test that is dependent on the hippocampus, amygdala and prefrontal cortex circuitry [62,63]. Interestingly, we observed that all treatments (combination, captopril, and atorvastatin) resulted in accelerated extinction in the fear conditioning test. This finding was independent of fear acquisition and tone conditioning which did not differ between the groups. Our data are consistent with previous studies using the angiotensin receptor type 1 antagonist losartan which increased extinction in adult mice [64,65,66]. Administration of losartan results in changes in gene expression in the amygdala that may underlie the observed effects on extinction behavior [64,65]. However, fear extinction is mediated by several brain areas, including the basolateral amygdala [67] as well as hippocampal-prefrontal cortex networks [68], and depends on glutamatergic [69] and BDNF signaling pathways [70]. A novel finding of our study is that all treatments, the combination, and either captopril or atorvastatin, increased extinction. A role for statins in extinction of fear conditioning has not been previously described. Our data suggest that these compounds may have a therapeutic role in psychiatric conditions such as PTSD in which aversive memories persist [64,65].

Overall, our research shows that combined treatment with atorvastatin and captopril has an anxiolytic effect in middle-aged mice, that may be mediated by a modest increase in adult neurogenesis. In adult mice, combination treatment as well as each compound separately increased fear memory extinction. Altogether, these drugs may regulate mood and adult hippocampal neurogenesis, and may have benefits for human patients with anxiety disorders.

## Figures and Tables

**Table 1:** Neurogenesis and angiogenesis in adult and middle-aged mice.

	Adult		Middle-Aged	
	Control	Combo	Control	Combo
**BrdU^+^ cells**	1071.6 (±57.4)	964.0 (±52.5)	683.2 (±39.0) **	869.3 (±29.5)
**BrdU^+^/NeuN^+^ cells (%)**	86.6 (±2.4)	85.5 (±2.3)	46.4 (±2.4) **	55.9 (±3.2) **
**Blood vessels (%)**	4.9 (±0.5)	4.8 (±0.4)	5.4 (±0.5)	5.1 (±0.3)

Middle-aged control mice had significantly less BrdU^+^ cells and a lower percentage of BrdU^+^/NeuN^+^ cells than all other groups. In the middle-aged mice, but not the adult group, combo treatment significantly increased the number of BrdU^+^ cells and the percentage of BrdU^+^/NeuN^+^ cells. Dentate gyrus blood vessel density did not differ between groups. ** significantly different from all other groups; *p* < 0.05.

**Table 2:** Open field behavior in adult mice.

Group	Total Distance (cm)	Center Time (s)	Periphery Time (s)
Control	9334.9 (±465.1)	858.1 (±112.1)	2741.8 (±112.1)
Combo	9588.1 (±510.3)	699.5 (±80.1)	2900.4 (±80.1)
Atorvastatin	8572.6 (±656.6)	633.7 (±75.6)	2966.2 (±75.6)
Captopril	8933.9 (±470.5)	706.8 (±87.6)	2893.1 (±87.6)

There was no difference between the groups in total distance travelled or center time as a result of combined or individual compound treatment in adult mice.

## Data Availability

The data presented in this study are available on request from the corresponding author.
